# The role of Ephexin1 in translation and mTOR-targeted cancer therapy

**DOI:** 10.1038/s12276-025-01520-2

**Published:** 2025-08-25

**Authors:** Jeeho Kim, In-Youb Chang, Jung-Hee Lee, Jeongsik Yong, Young Jin Jeon, Ho Jin You

**Affiliations:** 1 Laboratory of Genomic Instability and Cancer therapeutics, Chosun University School of Medicine, Gwangju, South Korea; 2 Department of Pharmacology, Chosun University School of Medicine, Gwangju, South Korea; 3 Department of Anatomy, Chosun University School of Medicine, Gwangju, South Korea; 4 Department of Cellular and Molecular Medicine, Chosun University School of Medicine, Gwangju, South Korea; 5 Department of Biochemistry, Molecular Biology and Biophysics, University of Minnesota Twin Cities, Minneapolis, MN USA

**Keywords:** Cancer models, Oncogenes

## Abstract

Ephexin1, also known as neuronal guanine nucleotide exchange factor (NGEF), plays a key role in axon guidance and synaptic homeostasis. However, our recent studies have revealed a critical role for Ephexin1 in the pathogenesis of colon and lung cancers. Here we used multidisciplinary approaches to further explore the underlying mechanisms of Ephexin1 in cancer progression. We discovered that Ephexin1 is essential for promoting polysome formation by coordinating the assembly of translation initiation complexes. Our investigations into gene expression affected by Ephexin1 deficiency showed that Ephexin1 specifically promotes the translation of genes containing 5′-terminal oligopyrimidine (TOP) or 5′-TOP-like motifs, identifying Ephexin1 as a key mediator of mTOR-regulated translation. Importantly, we found that the efficacy of mTOR inhibitors in treating lung cancer was significantly enhanced in a mouse xenograft model when Ephexin1 was deficient. This suggests that Ephexin1 could serve as a synthetic lethality target for mTORC1-targeting therapeutics in cancer treatment. Our findings provide mechanistic insights into the role of Ephexin1 in cancer pathogenesis and highlight its potential as a therapeutic target for improving current cancer treatment strategies.

## Ephexin1 drives cancer progression through mTOR regulation

Cancer cells grow faster than normal cells, needing more energy and nutrients. This study explores a protein called Ephexin1, which is often found in high levels in certain cancers. The authors used various laboratory techniques to study how Ephexin1 interacts with other proteins involved in starting protein production in cells. They found that Ephexin1 helps control the production of specific proteins that are important for cancer cell growth. This process is linked to the mTOR pathway. The study showed that reducing Ephexin1 levels made cancer cells more sensitive to mTOR-blocking drugs, suggesting a new way to enhance cancer treatment. The researchers conclude that targeting Ephexin1 could improve the effectiveness of existing cancer therapies and reduce side effects. Future research could focus on developing treatments that specifically target Ephexin1 to help fight cancer more effectively.

This summary was initially drafted using artificial intelligence, then revised and fact-checked by the author.

## Introduction

Cancer cells proliferate more rapidly than normal cells, necessitating increased metabolic inputs such as energy, amino acids, nucleotides and lipids. The mammalian target of rapamycin (mTOR) signaling pathway centrally orchestrates cell proliferation and growth^1,2^. Dysregulation of this pathway is implicated in numerous human diseases, underlining the importance of understanding mTOR-driven tumorigenesis as a crucial objective in cancer therapy^3–6^. However, challenges remain, particularly concerning the side effects and resistance associated with mTOR inhibitors, highlighting gaps in our understanding of the molecular mechanisms underlying effective treatment strategies for mTOR-driven tumors^7,8^.

mTOR functions through two distinct complexes, mTORC1 and mTORC2, which regulate diverse cellular processes^1^. mTORC1 primarily controls translation and autophagy, promoting cell growth, while mTORC2 influences cytoskeletal dynamics, cell survival and migration. Raptor and Rictor are key components of mTORC1 and mTORC2, respectively, essential for their function and localization^2^. Specifically, Raptor facilitates mTORC1 substrate recognition and ribosomal association, critical for mTORC1 assembly and its main function regulation of mRNA translation. mTORC1 modulates protein synthesis through phosphorylation of targets such as S6K1 and 4EBP1 and selectively regulates the translation of mRNAs containing 5′-terminal oligopyrimidine (TOP) motifs or 5′-pyrimidine-rich translational elements^2,9,10^.

The regulation of TOP mRNA translation by mTORC1 remains a promising but poorly understood area, suggesting potential strategies for cancer cell control^11^. Recent studies have highlighted roles for U-rich RNA-binding proteins such as LARP1, AUF1, TIA1 and TIAR in post-transcriptional regulation, though findings on their involvement in TOP mRNA translation are mixed and sometimes contradictory, indicating the complexity of this regulation^9,12–16^.

Translation initiation factor 3 (eIF3) plays a central role at the intersection of mTOR and S6K signaling pathways^17,18^ and is upregulated in various cancers^19,20^. As the largest and most complex of the eIFs, eIF3 consists of five essential core subunits (eIF3a, b, c, i and g) and seven additional subunits (eIF3d, e, f, h, k, l and m)^21^. Traditionally, eIF3 was thought to be released from the ribosome once the 40S and 60S subunits joined during translation^22^. However, recent studies have shown that eIF3 remains associated with the 60S ribosome, as well as with eukaryotic elongation factors (eEF) and transfer RNA synthetase^23–25^. In particular, the eIF3b-g-i subunit module acts like a mechanical arm that can adopt multiple conformations. After the 40S ribosome completes AUG scanning, a conformational change in this module prevents the release of eIF3 from the 80S ribosome and allows it to bind to the 60S subunit, promoting premature translation elongation^24,26,27^.

Ephexin1, a member of the Dbl family of guanine nucleotide exchange factors (GEFs), is typically less expressed outside the nervous system^28–30^. Associated with EphA receptors, Ephexin1 has primarily been studied in neurons, focusing on axon outgrowth, synaptic remodeling and motor axon guidance^29,30^. Research shows that Ephexin1 is often overexpressed in colon, lung and thyroid cancers. Recent studies indicate that Ephexin1 promotes the formation of EphA2–EGFR heterodimers, binds directly to Ras and increases malignancy, thereby worsening patient prognosis via the Ras–AKT signaling pathway^31–34^. Similarly, mTOR, which is also upregulated through the Ras–AKT pathway, plays a significant role in the development of malignant tumors^33,35^. Despite the critical roles of both Ephexin1 and mTOR signaling in cancer progression, their functional relevance remains largely unexplored.

In this study, we discovered that Ephexin1 directly binds to translation initiation factors 2α, 3g and 3i, playing a crucial role in cap-dependent translation. Notably, the interaction of Ephexin1 with the mTOR signaling pathway appears to regulate the translation of TOP/TOP-like mRNAs. Furthermore, we observed that the expression level of Ephexin1 correlates with the sensitivity to mTOR inhibitors; especially, combining Ephexin1 deficiency with mTOR inhibition significantly enhances the inhibitory effects on lung cancer cell proliferation. These findings provide new insights into the mechanism of translation control mediated by mTORC1 in lung cancer cells and suggest potential therapeutic strategies targeting these molecular interactions.

## Materials and methods

### Cell culture and transfection

H1299 were grown in RPMI-1640 medium (Invitrogen). LS174T cells were cultured in minimum essential medium. HEK-293T, SW480, SW620, DLD-1, U2OS, LoVo, HCT116, HCT116 p53^−/−^ and HT-29 cells were maintained in Dulbecco’s modified Eagle medium (Invitrogen). All cell lines were purchased from the American Type Culture Collection (ATCC). All media were supplemented with 10% fetal bovine serum (FBS) and 1% penicillin/streptomycin antibiotic solution. Cells were maintained in 5% CO_2_ in a humidified atmosphere at 37 °C. Plasmids were transiently transfected into mammalian cells using TurboFect (Thermo Scientific). Sodium arsenite, MK2206, SP600125, SB202190 and U0126 were purchased from Sigma-Aldrich and Torin1 and Bafilomycin A1 were purchased from MedChemExpress.

### Plasmid constructs and cloning

Human Ephexin1 was amplified by PCR from pCI-Flag-Ephexin1^33^. To prepare constructs of human Ephexin1 the PCR products were cloned into the BamHI sites of pEF1a-FLBIO-neo-vector^36^. Constructs were verified by DNA sequencing. For the isolation of recombinant proteins, the GST-Ephexin1 (full-length) construct was as previously described^33^. pEF1a-VirA-neo (no. 100548) and pFR_HCV_xb (no. 11510) plasmids were obtained from Addgene. A comprehensive list of all PCR primers used in this study can be found in Supplementary Table 3.

### RT–qPCR

Total RNA was extracted from cell lysates using TriZol (Invitrogen) and 2 μg of total RNA was reverse transcribed to cDNA using an oligo dT primer and M-MuLV Reverse Transcriptase (Invitrogen). Quantitative PCR with reverse transcription (RT–qPCR) analysis was performed using specific primers and the SYBR Premix Ex Taq kit (TaKaRa Bio). The transcripts were detected by CFX96 Real-Time PCR Detection System (Bio-Rad). The primers used for RT–qPCR were Ephexin1, HSP90ab1, eEF1α, C-myc, CCT2, β-actin and GAPDH. Each sample was analyzed in triplicate and target genes were normalized relative to the reference housekeeping gene, β-actin. Relative mRNA expression levels were calculated using the comparative threshold cycle (C_t_) method with β-actin as the control, according to the formula: ΔC_t_ = C_t_ (β-actin) − C_t_ (target gene). The fold change in gene expression normalized to β-actin and relative to the control sample was calculated as ΔΔC_t_. RT–qPCR primer sequences are listed in Supplementary Table 3.

### RNA interference

Cells were transfected with siRNAs (40 nM) using Lipofectamine 2000 (Invitrogen). After 36 h, cells were trypsinized, replated and transfected again for another 36 h. Knockdown efficiencies were verified by western blot analysis. Ephexin1 siRNA (1–3) and shRNA constructs were as previously described^33^, and siRNA and DNA oligonucleotides were synthesized by Macrogen. The sequences of Ephexin1 siRNA and shRNA can be found in Supplementary Table 4.

### Immunoblot and IP analysis

Cell extracts were prepared in immunoprecipitation (IP)150 lysis buffer (20 mM Tris–HCl pH 7.6, 150 mM NaCl, 0.5% Nonidet P-40 and 10% glycerol) containing protease inhibitors (1 mM Na_2_VO_4_, 10 mM NaF, 2 mM PMSF, 5 μg/ml leupeptin, 10 μg/ml aprotinin and 1 μg/ml pepstatin A) (Roche). Equal amounts of protein were separated by SDS–PAGE and transferred onto PVDF membranes (PALL Life Sciences). Membranes were subsequently incubated with the appropriate primary antibodies overnight at 4 °C, followed by incubation with peroxidase-conjugated secondary antibodies for 1 h at room temperature. Protein bands were visualized using the ECL chemiluminescent detection system (iNtRON Biotechnology). For IP of protein complexes, cell extracts were precleared with protein G-Sepharose beads (GE Healthcare) and incubated with the appropriate antibodies. Immune complexes were analyzed by immunoblotting using antibodies. The list of antibodies can be found in Supplementary Table 5.

### Tumor formation in nude mice

The mice used in this study were 6-week-old male BALB/c nude mice purchased from NARA Biotech. They were housed in our pathogen-free facility and handled in accordance with standard-use protocols and animal welfare regulations. H1299 cells were collected and resuspended in PBS. There after 1 × 10^6^ H1299 cells were injected subcutaneously into the left and right flanks of the mice. Once the tumors became visible, the tumor size was measured every 3 to 4 days using micrometer calipers. Tumor volumes were calculated using the following formula: volume = 0.5 *a* × *b*^2^, where *a* and *b* represent the larger and smaller tumor diameters, respectively. After approximately 8 weeks of injections, mice were humanely sacrificed, and the primary tumors were excised and immediately weighed.

### IHC

Immunohistochemistry (IHC) was performed on tissue microarrays of colorectal cancer samples. Tissue microarrays from cancer samples of different grades and adjacent normal tissues were purchased from Super Bio Chips (CCA4) and Biomax (LC483). For IHC, heat-induced antigen retrieval was performed using 1× antigen retrieval buffer (pH 9.0) (Abcam) at 95 °C for 15 min. After quenching of endogenous peroxidase and blocking in 3% H_2_O_2_ solution, tissues were incubated with primary antibodies overnight at 4 °C, followed by incubation with HRP-conjugated secondary antibody for 1 h at room temperature and incubation for 2 min in 3,3′-diaminobenzidine. The slides were then counterstained by introducing Harris’s hematoxylin. The intensity of staining was scored from 0 to 4 and the extent of staining was scored from 0% to 100%. The final quantitation score for each stain was obtained by multiplying the two scores. The slides were analyzed by two independent pathologists. The list of antibodies can be found in Supplementary Table 5.

### Polysome profiling analysis

Cell lysates of HEK-293T and H1299 were prepared in polysome profiling buffer (20 mM HEPES (pH 7.6), 125 mM KCl, 5 mM MgCl2, 2 mM dithiothreitol (DTT) and diethylpyrocarbonate-treated water) for sucrose gradient centrifugation. Extracts were incubated on ice for 15 min, and the insoluble material was pelleted by centrifugation at 13,000 rpm for 15 min. The resulting supernatant extracts were then loaded onto a ~17.5–50% sucrose gradient prepared with polysome profiling buffer and ultracentrifuged for 2.4 h at 35,000 rpm in an SW41-Ti rotor (Beckman). Post-centrifugation, the gradients were fractionated using a fraction collector (Brandel), and their quality was monitored at 253 nm using a UA-6 absorbance detector (Isco).

### Luciferase reporter assay

Cells seeded in 12-well plates were transiently transfected with luciferase reporter and pCI-Flag-Ephexin1 plasmids. Luciferase activity was determined with a dual-luciferase assay system (Promega). The activity was determined using a Glomax 20/20 luminometer (Promega).

### m7GTP pulldown assay

For the 7-monomethyl guanosine (m7GTP)-pulldown assay, cell extracts were precleared with protein A-agarose beads (Santa Cruz Biotechnoloy Inc.) and incubated with the m7GTP-agarose beads (Jena Biosciences) or protein A-agarose beads (control). m7GTP-agarose beads were equilibrated in m7GTP lysis buffer (50 mM HEPES pH 7.6, 100 mM KCl, 1 mM EDTA, 1 mM DTT, 0.5% NP-40, 10% glycerol, 1 mM Na_2_VO_4_, 10 mM NaF, 2 mM PMSF, 5 μg/ml leupeptin, 10 μg/ml aprotinin and 1 μg/ml pepstatin A) for 30 min before use. The m7GTP/protein complexes were then analyzed by immunoblotting. The antibodies are listed in Supplementary Table 5.

### Identification of genes related to sensitivity to mTOR targeting agents

Datasets of human cancer cell lines were obtained from DepMap portal (https://depmap.org/portal/, version 23Q2). Data on drug responses to mTOR targeting agents, including Torin1, Torin2, Temsirolimus, Sirolimus, Deforolimus and Everolimus, were obtained from the drug sensitivity PRISM^37^(version 23Q2) file. Genome-wide CRISPR loss-of-function screening data used data from two large-scale CRISPR experiments (Chronos^38^ and CERES^39^). Gene effects were calculated via DEMETER2 (ref. ^40^) in DepMap. The *P* value obtained from the test was then converted to −log10 (*P* value) to score each gene. The R program and GraphPad Prism (GraphPad Software Inc.) were used for visualization.

### Two-dimensional electrophoresis

In the first-dimension analysis, 1 mg of protein was applied via electric focusing onto immobilized pH gradient strips with a nonlinear pH range of 3–10. For the second-dimensional separations, the iso-electrically focused strips were electrophoresed on a 9–16% gradient polyacrylamide gel until the dye front reached the bottom of the gel. Subsequently, the gels were stained using Coomassie Brilliant Blue G-250, scanned utilizing a GS-710 imaging densitometer from Bio-Rad, and subjected to analysis using Melanie 7 image analysis software from GE Healthcare to quantify the relative abundance of protein spots.

### Identification of proteins by LC–MS/MS

Liquid chromatography–tandem mass spectrometry (LC–MS/MS) analysis was performed through nano ACQUITY UPLC and LTQ Orbitrap mass spectrometer (Thermo Electron). The column used BEH C18 1.7 μm, 100 μm × 100 mm column (Waters, Milford). The mobile phase A for the LC separation was 0.1% formic acid in deionized water and the mobile phase B was 0.1% formic acid in acetonitrile. The chromatography gradient was set up to give a linear increase from 10% B to 40% B for 16 min, from 40% B to 95% B for 8 min, and from 90% B to 10% B for 11 min. The flow rate was 0.5 µl/min. For MS/MS, mass spectra were acquired using data-dependent acquisition with full mass scan (300–2,000 *m*/*z*) followed by MS/MS scans. Each MS/MS scan acquired was an average of one microscans on the LTQ. The temperature of the ion transfer tube was controlled at 275 °C and the spray was 2.3 kV. The normalized collision energy was set at 35% for MS/MS. The individual spectra from MS/MS were processed using the SEQUEST software (Thermo Quest) and the generated peak lists were used to query in house database using the MASCOT program (Matrix Science Ltd.). We set the modifications of carbamidomethyl (C), deamidated (NQ) and oxidation (M) for MS analysis and the tolerance of the peptide mass was 10 ppm. MS/MS ion mass tolerance was 0.8 Da, the allowance of missed cleavage was 2 and charge states (+2, +3) were considered for data analysis. We took only significant hits as defined by MASCOT probability analysis.

### Ribopuromycylation assay

HEK-293T and H1299 cells were seeded in a 60 mm plate and grown for 2 days. The cells were pulsed with 10 μg/ml puromycin 10 min at 37 °C in 5% CO_2_ incubator. The cells were washed with cold PBS twice and lysed with RIPA lysis buffer. All proteins were subjected to western blot analysis. Anti-puromycin antibody used to detect against ribopuromycylated proteins.

### RNP-IP

The protocol for ribonucleic acid–protein IP (RNP-IP) follows a previously described method^41^. Cells were washed with cold PBS and resuspended in lysis buffer (20 mM Tris–HCl, pH 7.6, 150 mM NaCl, 0.5% Nonidet P-40 and 10% glycerol) supplemented with protease inhibitors (1 mM Na_2_VO_4_, 10 mM NaF, 2 mM PMSF, 5 μg/ml leupeptin, 10 μg/ml aprotinin and 1 μg/ml pepstatin A) and 40 U/ml RiboLock RNase inhibitor (Fermentas) in diethylpyrocarbonate-treated water. After a 15 min incubation, samples were centrifuged at 13,500*g* for 15 min. The supernatants were then subjected to IP with streptavidin agarose (Invitrogen) at 4 °C overnight. Following IP, the beads were washed five times with lysis buffer and the RNA was extracted using Trizol. The RNA was then treated with DNase (TURBO DNA-free kit) to remove any contaminating DNA and subsequently used for RT–qPCR. Primer sequences for RT–qPCR are provided in Supplementary Table 3.

### Statistics

Data were presented as mean ± s.e.m. of three independent experiments and significant differences between groups were assessed by two-tailed paired Student’s *t*-test or two-way analysis of variance (ANOVA) using GraphPad Prism (GraphPad Software Inc.). Results with a value of *P < 0.05, **P < 0.01 and ***P < 0.001 were considered statistically significant.

## Results

### Ephexin1 interacts with translation factors

**Fig. 1:**
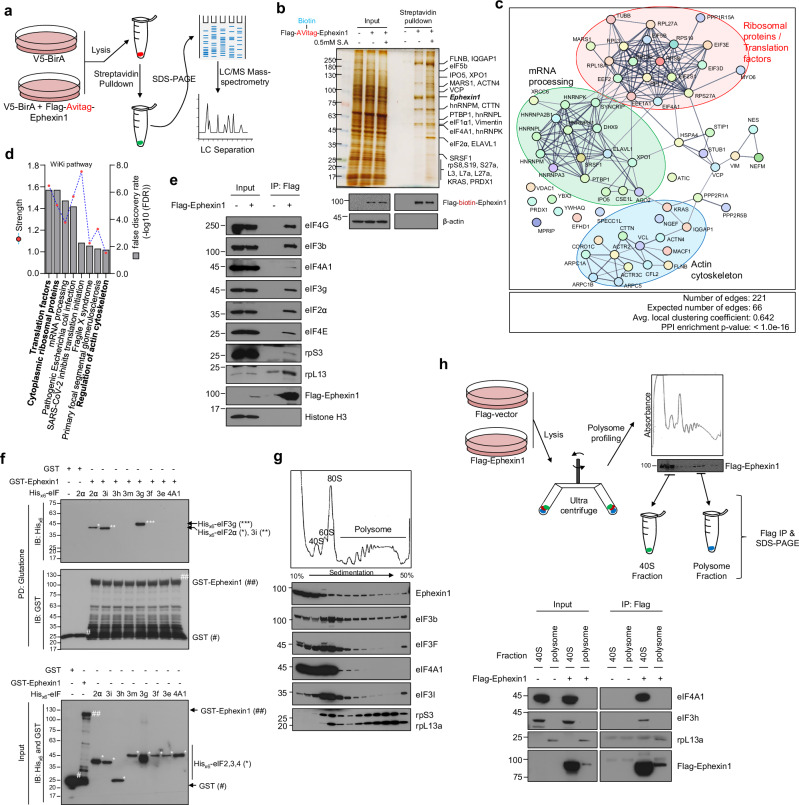
Ephexin1 is associated with translation initiation factors. **a** A schematic of the method used to detect biotinylated Ephexin1 via IP and MS. **b** A silver-stained gel showing the protein interactome of biotinylated Ephexin1. The analysis was performed on cells either untreated or exposed to sodium arsenite (0.5 mM for 40 min). **c** STRING analysis representing the Ephexin1 interactome. Proteins linked to Ephexin1 are organized by related biological pathways and grouped within a circle to highlight thematic relationships. **d** Wiki_Pathways visualization of the biological pathways implicated in the Ephexin1 interactome. **e** Validation of Ephexin1 interactome analysis. IP using anti-flag antibody was conducted on cell extracts from HEK-293T cells transfected with Flag-tagged Ephexin1. The precipitated proteins were then analyzed by western blot, employing specific antibodies to detect and confirm the presence of interacting proteins. **f** An in vitro GST-pulldown assay showing the interaction between recombinant His_x6_-tagged translation factors (eIF2α, eIF3i, eIF3h, eIF3m, eIF3g, eIF3f, eIF3e and eIF4A1) and GST or GST-tagged Ephexin1 recombinant protein. **g** Polysome profiling analysis of Ephexin1, various translation initiation factors and ribosomal proteins using H1299 cells. **h**, A schematic of the Ephexin1–translation factor interaction study. The diagram illustrates the co-IP experiments conducted using 40S ribosomal and polysome fractions isolated from HEK-293T cells transfected with Flag-tagged Ephexin1. Western blot analysis was performed using specific antibodies to detect and analyze the interactions between Ephexin1 and various translation factors. FDR, false discovery rate; PPI, protein-protein interaction; IB, immunoblotting; PD, pull-down assay.

Previous studies have demonstrated that Ephexin1 is upregulated in lung cancer and significantly contributes to its malignancy^33,34^. To further explore the physiological significance and underlying mechanisms of these findings, we engineered HEK-293T cells to stably express both Flag-tagged and biotinylated Ephexin1, enabling us to probe the Ephexin1 interactome (Fig. 1a). We performed pulldown assays using streptavidin on these engineered cells, as well as on control cells expressing only the BirA biotin-ligase (BirA/HEK-293T). MS analysis of the proteins specifically pulled down by biotinylated Ephexin1 identified 65 proteins implicated in various biological processes (Fig. 1b and Supplementary Table 1). A STRING-based clustering analysis of these proteins highlighted that Ephexin1 interacts not only with components of the previously documented actin cytoskeleton pathway^30^ but also with key players in translation initiation, ribosomal function and mRNA processing (Fig. 1c, d). To validate these interactions, we expressed Flag-tagged Ephexin1 in HEK-293T cells and examined its interactions with the identified proteins through co-IP and western blotting (Fig. 1e). In addition, arsenite and heat shock treatments, which induce aggregation of translation initiation factors, increased the interaction of Flag-tagged Ephexin1 with eIF3b, eIF4A1, eIF2a and eIF4E (Supplementary Fig. 1). These results confirmed the interactions between Ephexin1 and crucial components of the translation initiation machinery and ribosomal proteins, supporting the MS findings. To determine whether the co-IP interactions between Ephexin1 and translation initiation factors result from direct binding, we conducted binding experiments using recombinant GST-fused Ephexin1 and His_x6_-tagged translation initiation factors, as detailed in Fig. 1f. Our results showed that eukaryotic initiation factors 2α, 3g and 3i bind directly to Ephexin1 (Fig. 1f). This demonstrates that Ephexin1 is directly involved with translation initiation complexes. Notably, eIF3i/g is a core subunit of the eIF3 complex and is critical for its overall function^21^.

To investigate the role of Ephexin1 in translation, we used polysome profiling to analyze the localization of Ephexin1 in normal and EDTA-treated ribosome fractions (Fig. 1g and Supplementary Fig. 2). Intriguingly, polysome profiling revealed that Ephexin1 predominantly associates with the 40S and 60S ribosomal peaks, indicating its specific involvement in early stages of translation. To further elucidate the interactions of Ephexin1 with translation initiation factors, we performed co-IP experiments using polysome fractions isolated from HEK-293T cells expressing either control or Flag-tagged Ephexin1. These experiments confirmed that Flag–Ephexin1 interacts with key translation initiation factors, such as eIF4A1 and eIF3s, specifically within the 40S ribosomal fractions (Fig. 1h). These findings confirm the association of Ephexin1 with translation initiation factors in the cytoplasm and provide compelling evidence that Ephexin1 plays an active role in the translation initiation process.

### Ephexin1 promotes cap-dependent translation initiation

**Fig. 2:**
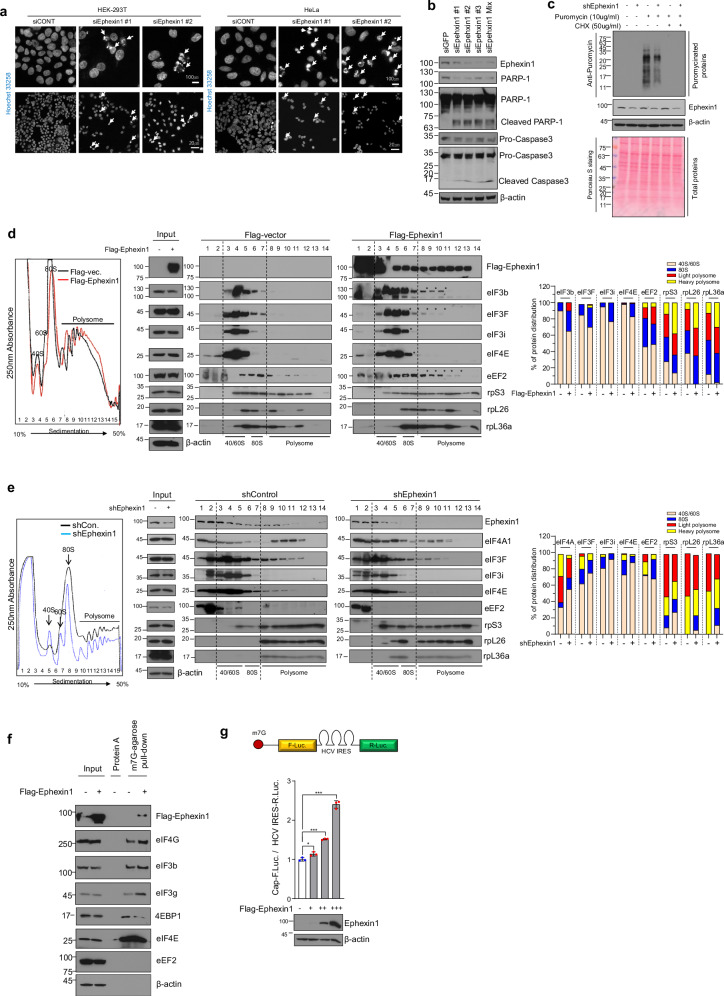
Ephexin1 promotes cap-dependent translation initiation. **a** HEK-293T and HeLa cells were transfected with siCONT or siEphexin1 (nos. 1–2) and nuclear staining was performed using Hoechst 33258. The white arrows indicate cells with fragmented nuclei. **b** siControl and siEphexin1 were transfected into H1299 cells for 72 h and analyzed by western blot to detect protein levels. **c** Translation efficiency in response to Ephexin1 depletion was evaluated using a ribopuromycylation assay in HEK-293T cells subjected to either control treatment or Ephexin1 knockdown. The extent of puromycylated proteins was quantified by western blot analysis using an anti-puromycin antibody, while total protein levels were assessed through Ponceau S staining. **d** The distribution of Ephexin1 and selected translation factors in the polysome profiles of HEK-293T cells transfected with either a Flag-empty vector or Flag-tagged Ephexin1 was analyzed. **e** The distribution of translation factors in the polysome profiles upon the knockdown of Ephexin1 in HEK-293T cells. **f** The role of Ephexin1 in assembling eIF4F and eIF3 complexes. The impact of Ephexin1 on the formation of eIF4F and eIF3 complexes was investigated using an m7GTP pulldown assay. HEK-293T cells transfected with either a Flag-empty vector or Flag-tagged Ephexin1 were analyzed. eEF2 served as a negative control. Proteins associated with the m7G cap were identified through western blot analysis. **g** The influence of Ephexin1 on cap-dependent translation initiation was examined in HEK-293T cells cotransfected with Flag-tagged Ephexin1 and an FLuc-HIV_IRES-RLuc plasmid. Translation activity was assessed using a dual-luciferase assay.

Cancer cell proliferation, migration and invasion rely heavily on continuous protein synthesis^42^. Previous studies have demonstrated that Ephexin1 deficiency impedes the growth of lung cancer and colorectal cancer^33^. Furthermore, translational repression has been linked to the induction of apoptosis^43^, a response we have observed in cells lacking Ephexin1 (Fig. 2a,b). To investigate whether the apoptosis associated with Ephexin1 deficiency is linked to its role in translational regulation, we knocked down Ephexin1 in H1299 and HEK-293T cells and performed ribopuromycin analysis to assess new protein synthesis. The results, illustrated in Fig. 2c, reveal that Ephexin1 knockdown significantly reduces puromycinated proteins compared to controls, underscoring the crucial role of Ephexin1 in maintaining normal protein synthesis mechanisms in cancer cells (Fig. 2c and Supplementary Fig. 3a).

Overexpression of Flag-tagged Ephexin1 in HEK-293T cells led to an increase in polysome formation, as observed through polysome profiling analysis. This overexpression also promoted the movement of several key factors—including eIF3F, eIF3b, eIF3i, eIF4E and eEF2—into the polysome fraction, along with ribosomal proteins for both small and large ribosomes (Fig. 2d). These findings align with previous studies suggesting that certain translation initiation factors, such as eIF3, not only facilitate translation initiation but also contribute to translation elongation by staying associated with the 80S ribosome and elongation factors^23–27^. In contrast, when Ephexin1 was depleted in HEK-293T and H1299 cells, a reduction in polysome area was observed during polysome profiling, along with prominent peaks for the 40S and 60S ribosomal subunits. Additionally, this depletion caused the movement of eIF4A1, eIF4E, eIF3b, eIF3F, eIF3i and eEF2 into monosomes (Fig. 2e and Supplementary Fig. 3b,c). These results strongly suggest that Ephexin1 plays a key role in regulating translation within cells.

Translation initiation is a critical regulatory step in protein synthesis, influenced by various cellular pathways and protein factors^44^. To investigate the functional significance of Ephexin1’s interaction with components of the translation initiation complex, we assessed its effect on the assembly of these complexes on the m7G cap structure of mRNAs. In HEK-293T cells overexpressing Flag-tagged Ephexin1, pulldown analysis using m7G cap analog-conjugated agarose beads revealed significant findings. Overexpression of Ephexin1 enhanced the association of eIF4G, eIF3b and eIF3g with the m7G cap, whereas the binding of 4EBP1 to the m7G cap was notably reduced (Fig. 2f). Ephexin1 deficiency consistently diminished the interaction between eIF4A1 and eIF3i with the m7G cap, a finding paralleled in IP analyses using eIF4E antibodies (Supplementary Fig. 4a–c). These observations suggest that Ephexin1 overexpression promotes the formation and activation of initiation complexes, leading to enhanced translation initiation by reducing the association of 4EBP1 with the mRNA cap structure. Furthermore, these effects on translation regulation by Ephexin1 may be linked to mTOR signaling, as the dissociation of 4EBP1 from the cap structure is known to be stimulated by mTOR activation^1,2^. To verify that Ephexin1 overexpression enhances translation initiation complex formation and subsequently increases protein synthesis, we utilized a luciferase assay to measure translation activity. The results depicted in Fig. 2g demonstrate that cap-dependent translation, as indicated by luciferase activity, increases in direct correlation with the expression levels of Flag–Ephexin1 (Fig. 2g). These findings collectively confirm that Ephexin1 not only facilitates the assembly of initiation complexes but also significantly boosts translation in cells.

### Translational regulation of Ephexin1 is associated with the mTORC1 pathway

**Fig. 3:**
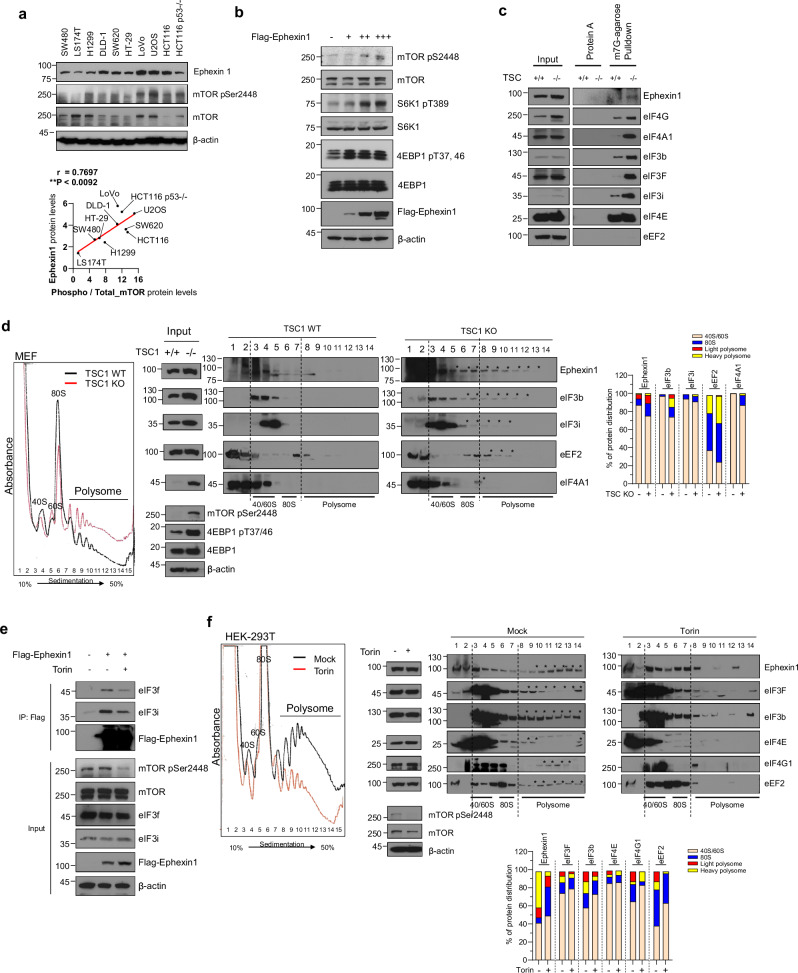
Translation regulation by Ephexin1 is associated with mTOR signaling. **a** Correlation analysis of Ephexin1 expression and mTOR kinase phosphorylation across ten cancer cell lines. Western blot analysis was conducted to evaluate the relationship between Ephexin1 expression levels and the phosphorylation status of mTOR kinase in ten different cancer cell lines using the specified antibodies. **b** Activation of mTOR signaling in response to Ephexin1 overexpression was assessed by measuring the phosphorylation levels of downstream target proteins. **c** Ephexin1, eIF4F and eIF3 complexes were isolated from the lysates of wild-type MEF (MEF WT) and TSC knockout (TSC KO) cells using an m7GTP pulldown assay. The composition of these complexes was subsequently analyzed by western blot. **d** The protein distribution in polysome profiling of TSC1 WT and TSC1 KO MEF cells. Protein profiles from polysome profiling of both TSC1 WT and TSC1 KO MEF cells were analyzed by western blot using the specified antibodies. **e** Co-IP was conducted on protein extracts from HEK-293T cells transfected with Flag-tagged Ephexin1 and treated with Torin1 (250 nM for 1 h). IP was performed using an anti-Flag antibody. **f** Polysome profiling was conducted on HEK-293T cells treated either with a mock solution or Torin1 (250 nM for 1 h). The distribution of proteins was analyzed using western blot with the specified antibodies.

The mTORC1 signaling pathway is crucial for controlling translation^2^. Based on evidence that Ephexin1 influences translational activity and its expression correlates with the phosphorylation status of 4EBP1 (Fig. 2 and Supplementary Fig. 5a,b), we hypothesized that Ephexin1 plays a role in mTORC1-mediated translation regulation. To explore this hypothesis, we examined the relationship between Ephexin1 expression and mTOR activation across various cancer cell lines. There are seven recognized phosphorylation sites on mTOR, among which the phosphorylation at Ser2448 is crucial for the activity of mTORC1 (refs. ^7,45^). Western blot analysis revealed a strong correlation between Ephexin1 expression and phosphorylation of mTOR at Serine 2448 (*r* = 0.7697, *P* = 0.0092), suggesting that Ephexin1 is significantly linked to mTOR activation (Fig. 3a). To further investigate whether increasing Ephexin1 levels enhances cellular mTOR activity, we elevated Ephexin1 expression through transient transfection and monitored the phosphorylation status of mTOR and its downstream targets, S6K1 and 4EBP1. The results depicted in Fig. 3b show that as Ephexin1 expression increased, there was a corresponding increase in the phosphorylation of mTOR, S6K1, and 4EBP1, underscoring the importance of Ephexin1 in the activation of mTOR signaling pathways. Depletion of *Tsc1*, a negative regulator of mTORC1, led to mTORC1 hyperactivity^46,47^. In *Tsc1* knockdown MEF cells, there was increased recruitment of Ephexin1, eIF4F complexes, and eIF3 complexes to the 5’ mRNA cap compared to wild-type cells (Fig. 3c). Polysome profiling further showed that *Tsc1* knockout caused Ephexin1, eIF4A1, eIF3b, eIF3i, and eEF2 to shift from monosomes to polysomes, in contrast to wild-type cells (Fig. 3d). Conversely, treatment with the mTOR inhibitor Torin1 in HEK-293T cells overexpressing Flag–Ephexin1 reduced the interaction between Flag–Ephexin1 and eIF3f/eIF3i (Fig. 3e). Consistent with this, Torin1 treatment caused Ephexin1, eIF4F complexes, eIF3 subunits, and eEF2 to shift from polysome to monosome in polysome profiling analysis (Fig. 3f). These results suggest a potential link between Ephexin1 and mTOR signaling in the regulation of translation.

### Ephexin1 modulates translation of TOP/TOP-like mRNAs

**Fig. 4:**
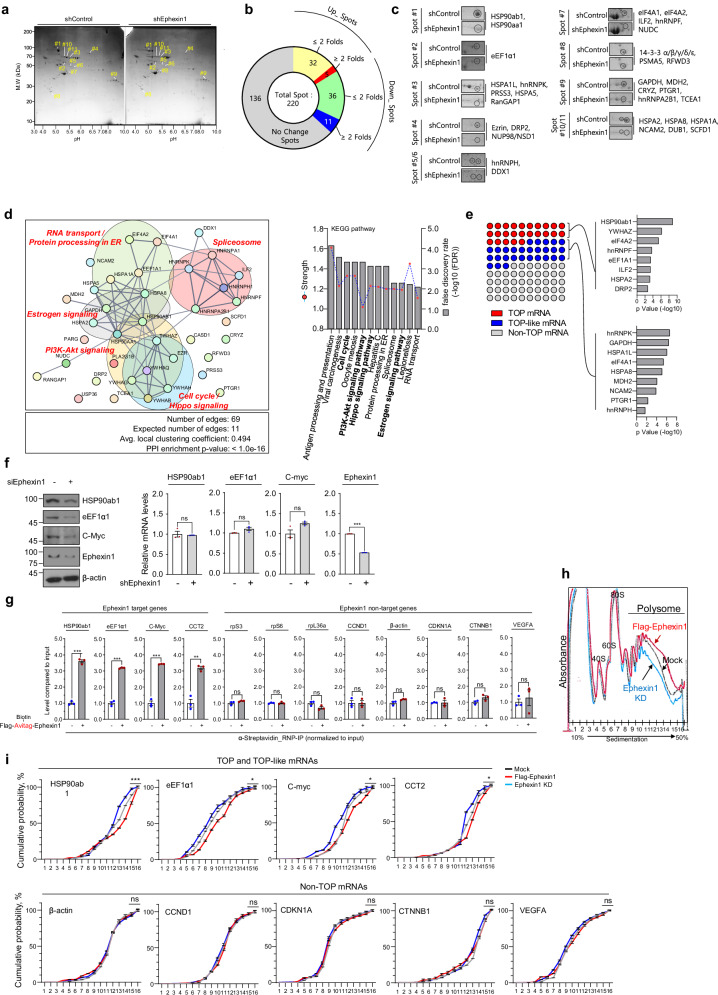
Regulatory role of Ephexin1 in translation initiation is associated with an mTORC1-controlled translation mechanism. **a** The 2D gel electrophoresis analysis of proteins upon the knockdown of Ephexin1 in H1299 cells. The yellow numbers highlight the protein spots reduced by more than twofold in Ephexin1-deficient extracts. **b** Comparative protein expression analysis in shControl and shEhexin1_H1299 cells using 2D gel electrophoresis image analysis. **c** Magnified images and MS of 11 spots showing more than a twofold reduction in the previous 2D-gel electrophoresis. **d** Proteins exhibiting reduced expression in Ephexin1-deficient cells were analyzed using STRING to determine interactions and functional associations. Cluster analysis displays strength and false discovery rate (FDR) values for protein clusters showing decreased levels in Ephexin1-deficient lysates. **e** Identification of TOP/TOP-like feature containing genes among proteins identified in the 2D gel and MS analyses. **f** Protein and mRNA levels of genes identified in **e** in H1299 cells. **g** RNP-IP analysis using RT–qPCR after pulldown with streptavidin in a HEK-293T/Flag-biotinylated Ephexin1 stable cell line. **h**, **i** Polysome profiling upon gain or loss of Ephexin1 in HEK-293T cells: cells transfected with shEphexin1 or Flag–Ephexin1 underwent polysome fractionation (**h**), followed by RT–qPCR analysis of TOP/TOP-like and non-TOP feature containing mRNAs, presented in a cumulative plot (**i**). KEGG, Kyoto Encyclopedia of Genes and Genomes; ns, not significant.

To elucidate the role of Ephexin1 in translational regulation within cancer cells, we utilized two-dimensional (2D) gel electrophoresis and MS to identify proteins whose expression levels decrease as a result of Ephexin1 loss. This analysis was performed on control and Ephexin1-depleted H1299 cells (Fig. 4a). In our 2D gel electrophoresis study, we identified a total of 220 protein spots. Quantitative image analysis revealed that 47 of these spots exhibited reduced intensity due to Ephexin1 deficiency, with 11 of them showing a decrease of more than twofold. In contrast, 37 spots increased in intensity in Ephexin1-deficient cells, with five showing an increase greater than twofold. No significant changes were detected in the remaining 136 spots (Fig. 4b). Since Ephexin1 deficiency leads to translation inhibition (Fig. 2), we focused on the protein spots that were reduced following Ephexin1 depletion. Protein spots that exhibited more than a twofold change were subjected to further MS analysis. This led to the identification of 47 statistically significant protein peptides from the 11 reduced spots (Fig. 4c and Supplementary Table 2).

We conducted functional classification and network association analysis using STRING software (https://string-db.org) to categorize the downregulated proteins identified from 2D gel electrophoresis LC–MS/MS analysis. These proteins were linked to key pathways such as cell cycle regulation, PI3K–AKT signaling, Hippo signaling, protein processing, spliceosome and estrogen signaling (Fig. 4d). The downregulation of these pathways is known to inhibit cancer cell proliferation and is closely associated with mTOR signaling^35,48,49^. Notably, 53% of the proteins downregulated due to Ephexin1 deficiency in our analysis contained 5′-TOP or 5′-TOP-like motifs in their genes (Fig. 4e). Interestingly, ribosomal proteins, which are some of the most well-known 5′-TOP-regulated gene products^10^, were not detected in our MS analysis. Consistently, the protein levels of ribosome proteins S3, S6, L26 and L36a remained unchanged by either Flag–Ephexin1 overexpression or depletion (Supplementary Fig. 6). In contrast, Ephexin1 predominantly influenced the expression of cancer-associated proteins with 5′-TOP motifs in their genes (Fig. 4a–e and Supplementary Table 2). These proteins—such as HSP90ab1, eEF1α1, c-Myc and CCT2—play critical roles in cancer cell growth and survival by regulating protein stability, synthesis and transcription^50–55^. In Ephexin1-deficient cells, these proteins were downregulated, even though they are normally activated by mTOR through translational regulation of mTOR-responsive *cis*-elements^9,10^. As a result of Ephexin1 deficiency, the protein levels of HSP90ab1, eEF1α1 and c-Myc were reduced, although their mRNA levels remained unchanged. No changes in protein stability due to ubiquitination were observed (Fig. 4f and Supplementary Fig. 7a,b). Interestingly, in the biotinylated Ephexin1/HEK-293T cell line, streptavidin pulldown followed by RNP-IP analysis demonstrated an association between Ephexin1 and the mRNAs of HSP90ab1, eEF1α1, c-Myc and CCT2. In contrast, proteins not regulated by Ephexin1 exhibited no such association (Fig. 4g and Supplementary Fig. 8).

On the basis of these findings, we hypothesized that Ephexin1 regulates the translation of 5′-TOP mRNAs, which are under the control of the mTORC1 pathway. To test this hypothesis, we performed polysome profiling analysis in HEK-293T cells with Ephexin1 overexpression and depletion. Compared with control cells, Ephexin1 depletion caused the mRNAs of HSP90ab1, eEF1α1, c-myc and CCT2 to shift to lighter polysome fractions, indicating reduced translation. In contrast, overexpression of Ephexin1 shifted these mRNAs to heavier polysome fractions, signifying increased translation. Importantly, non-TOP mRNAs, such as β-actin, CCND1, CDKN1A, CTNNB1 and VEGFA, did not exhibit any changes in polysome distribution (Fig. 4h,i). These results suggest that Ephexin1 may serve as a novel mediator of mTORC1-regulated TOP mRNA translation.

**Fig. 5:**
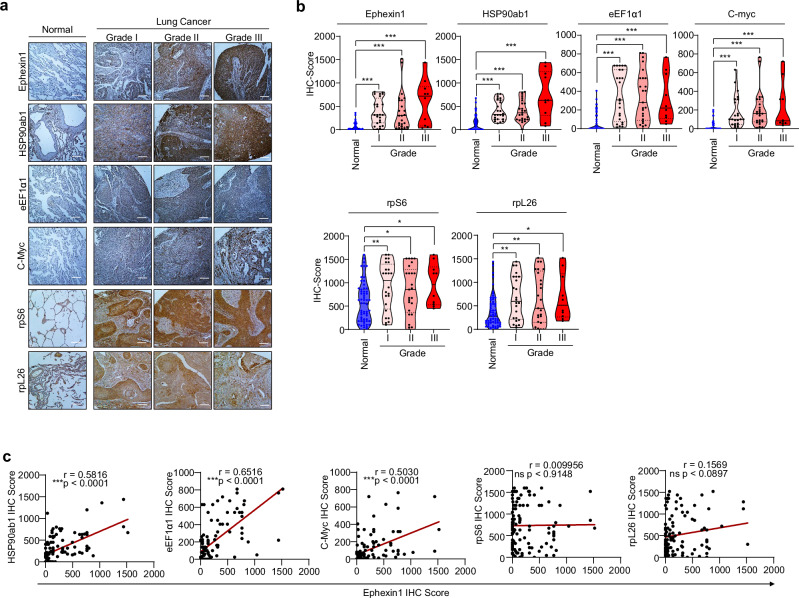
Expression of Ephexin1 and mTOR target proteins is correlated in patients with lung cancer. **a** Evaluation of Ephexin1, HSP90ab1, C-myc and eEF1α1 expression across different grades of lung cancer tissues (I (*n* = 24), II (*n* = 25) and III (*n* = 10)) and corresponding normal tissues (*n* = 59) using IHC staining. Hematoxylin was used as a counterstain. Scale bar, 100 μm. **b** Expression scores for Ephexin1, HSP90ab1, C-myc and eEF1α1 in lung tissues, analyzed via Student’s *t*-test. Data shown as mean ± s.e.m. Significance levels marked as ns (not significant), *P < 0.05, **P < 0.01 and ***P < 0.001. **c** A correlation of IHC scores between Ephexin1 and HSP90ab1, eEF1α1 or c-myc, shown as mean Spearman *r* values. *P* values are for a two-tailed Student’s *t*-test, with significant correlations indicated (*P* < 0.001).

In lung cancer, deregulation of mTOR signaling plays a key role in accelerating tumor progression and increasing malignancy^56,57^. Additionally, elevated Ephexin1 expression in lung cancer is closely linked to both tumor progression and patient prognosis^31,33^. On the basis of these observations, our findings on Ephexin1’s regulation of 5′-TOP mRNA translation, we hypothesized that mTOR target genes, such as HSP90ab1, eEF1a1 and c-Myc, along with Ephexin1, are involved in lung cancer progression. To explore the clinical relevance of these proteins in lung cancer, we conducted a tissue microarray analysis using samples that included normal lung tissues, various grades of carcinoma and metastatic tumors from patients with lung cancer Our findings revealed that the levels of Ephexin1, HSP90ab1, c-Myc and eEF1α1 were significantly elevated in lung cancer tissues compared with normal tissues and showed a progressive increase with advancing tumor grade and metastasis (Fig. 5a, b). Moreover, the expression levels of Ephexin1 and associated mTOR target genes were positively correlated, demonstrating statistical significance (Fig. 5c). However, ribosomal proteins S6 and L26 were elevated in tumors compared with normal tissues, but their correlation with Ephexin1 was not statistically significant (Fig. 5a–c).

### Role of Ephexin1 in enhancing mTOR inhibitor efficacy against cancer

**Fig. 6:**
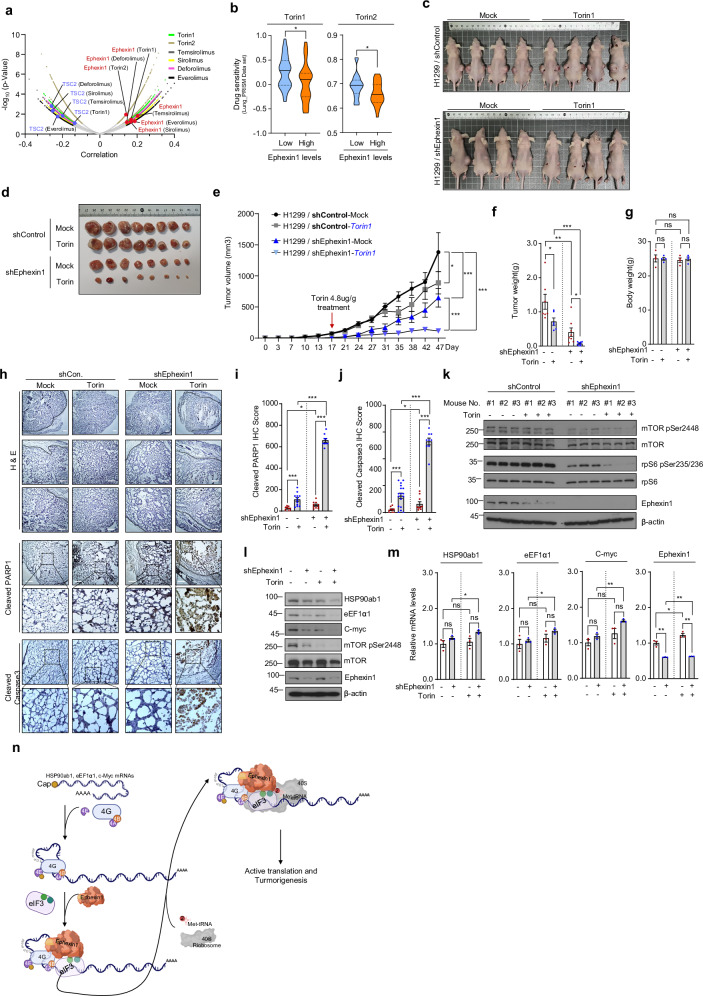
Enhanced anticancer efficacy of mTOR inhibitors in Ephexin1-deficient lung cancer. **a** A volcano plot illustrating the correlation between sensitivity to mTOR-targeted agents and gene effects, determined through CRISPR screening (DEMETER2). Ephexin1 is highlighted with a red circle, while TSC2, a negative regulator of mTOR, is marked with a blue circle. **b** Analysis of Torin1 and Torin2 drug sensitivity based on Ephexin1 mRNA expression levels in lung cancer cells (Torin1, *n* = 30; Torin2, *n* = 28). **c**, **d** Control and Ephexin1-depleted H1299 cells were subcutaneously transplanted into BALB/c nude mice (*n* = 6). Starting at 17 days post-transplantation, Torin1 (4.8 mg/g) was administered every 3 or 4 days. After 30 days of administration (**c**), mice were killed and tumors were collected (**d**). **e** Tumor volumes recorded at specified intervals are presented. Data are expressed as mean ± s.e.m. Statistical significance was determined using a two-way ANOVA: ***P < 0.001 and *P < 0.05. **f**, **g** At the end of the experiment, average tumor weights (**f**) and body weights (**g**) of each group were measured. Values are mean ± s.e.m. Statistical significance is denoted as ns (not significant), *P < 0.05, **P < 0.01 and ***P < 0.001, assessed with a two-tailed Student’s *t*-test. **h** Hematoxylin and eosin (H & E) staining along with IHC for cleaved PARP1 and cleaved Caspase3 of H1299 xenograft tumors. Scale bar, 100 μm. **i**, **j** Proliferation indices, based on cleaved PARP1 (**i**) and cleaved Caspase3 (**j**), in H1299 xenograft tumors. Data are presented as mean ± s.e.m., with *P < 0.05 and ***P < 0.001, using a two-tailed Student’s *t*-test. **k** The levels of phosphorylated mTOR and S6 in H1299 xenograft tumors were analyzed by western blot using specific antibodies. **l**, **m** Control and Ephexin1-deficient H1299 cells treated with Torin1 (250 nM for 1 h) were analyzed and protein levels (**l**) and mRNA levels (**m**) were assessed by western blot and RT–qPCR, respectively. **n** A schematic illustration of the proposed model for Ephexin1-mediated regulation of intracellular translation.

Torin1, Torin2, Temsirolimus, Sirolimus, Deforolimus and Everolimus are inhibitors that target the mTOR, a key kinase involved in cell growth, proliferation, and metabolism. These inhibitors are extensively utilized in both research and clinical settings for cancer treatment, immune modulation and in other diseases^4,6^. Despite considerable advances in developing these inhibitors as cancer therapies^4–7^, resistance to the drugs continues to pose significant challenges^58,59^. To explore this issue further, we investigated whether there is a link between the expression of Ephexin1 in cancer cells and their resistance to mTOR inhibitors. To further investigate this issue, we utilized datasets available on the DepMap portal (https://depmap.org/portal/, version 23Q2). We analyzed the viability of cancer cells in relation to Ephexin1 gene expression, which was modulated using CRISPR technology, following treatment with mTOR inhibitors^37–39^. Our findings revealed a strong positive correlation between decreased Ephexin1 expression and decreased viability of cancer cells treated with these inhibitors. Conversely, the *TSC2* gene, which is instrumental in suppressing mTOR signaling, showed a significant negative correlation. Notably, cancer cells demonstrated enhanced sensitivity to the potent mTOR inhibitors, Torin1 and Torin2, when Ephexin1 expression was reduced (Fig. 6a, b and Supplementary Fig. 9).

Further investigation into the role of Ephexin1 in cancer progression revealed that Ephexin1 deficiency influences mTORC1 signaling pathway activity, impacting tumorigenesis. In an experimental setup, Torin1 was administered every 3–4 days over 30 days to mice with tumors derived from either control H1299 cells or Ephexin1-deficient H1299 cells. Tumors in Ephexin1-deficient mice were notably smaller in both volume and weight compared with controls treated with Torin1, although body weights were not different (Fig. 6c–g). Additionally, immunohistological analysis of these tumors showed significantly higher levels of cleaved PARP1 and cleaved Caspase3 in the Ephexin1-deficient and Torin1-treated group compared to controls (Fig. 6h–j).

Immunoblot analysis further demonstrated that Ephexin1 depletion led to reduced phosphorylation of mTOR and its target protein rpS6, enhancing the inhibitory effects of Torin1 on mTOR signaling (Fig. 6k). Interestingly, in Ephexin1-deficient H1299 cells, Torin1 treatment did not alter—or even slightly increased—the mRNA levels of HSP90ab1, eEF1α and c-myc, but significantly reduced their protein levels (Fig. 6l, m). Consistently, treatment with the mTORC1-specific inhibitor Everolimus resulted in a significant reduction in cell viability in Ephexin1-deficient cells compared with the control group. Western blot analysis revealed similar results, akin to those observed following Torin treatment (Supplementary Fig. 10a, b). These findings collectively suggest that targeting Ephexin1 may potentiate the tumor-suppressive effects of mTOR inhibitors in lung cancer therapy.

## Discussion

Ephexin1, also known as neuronal guanine nucleotide exchange factor (NGEF), is predominantly expressed in the nervous system, where it plays a key role in axon guidance and synaptic homeostasis^29,30^. Dysregulation of Ephexin1 has been linked to various neurological conditions, including neurodevelopmental and neurodegenerative disorders, as well as depression^29,30,60^. Interestingly, the initial study that identified NGEF also revealed its oncogenic potential by showing that H-Ras^G12V^ overexpression increases Ephexin1 mRNA levels^28,61^. Despite this early finding, the role of Ephexin1 in cancer biology was largely overlooked. Recent research, including our own, has highlighted its oncogenic function, particularly in colon and lung cancers^31–34^. Ephexin1 deficiency significantly inhibits the growth of these cancers and is strongly correlated with patient prognosis^33,34^.

In this study, we explored the molecular mechanisms underlying the role of Ephexin1 in cancer pathogenesis. We discovered that Ephexin1 is crucial for regulating cap-dependent translation initiation, specifically focusing on 5′-TOP motif-containing genes, which are highly relevant in cancer progression. While mTOR-regulated translation of 5′-TOP mRNAs is well documented, the underlying molecular mechanisms are not fully understood^9–11^. Our findings identify Ephexin1 as a key interface between mTOR signaling and the translation of 5′-TOP mRNAs. This regulatory role is closely linked to the prognosis of patients with lung cancer, where Ephexin1 overexpression is associated with increased cell proliferation, migration and overall prognosis^33,34^.

mTOR is frequently deregulated in many cancers, making it a critical target for cancer therapies^4–6^. Rapalogs, rapamycin analogs, have been approved for treating advanced cancers such as renal cell carcinoma, pancreatic neuroendocrine tumors and advanced breast cancer^62–64^. However, their therapeutic effectiveness can be compromised by feedback activation of IGF-IR and AKT, counteracting the effects of mTORC1 inhibition^65^. Drug resistance also remains a significant challenge in cancer treatment, as long-term use of chemotherapy and targeted therapies often leads to resistance. Tumors with KRAS, BRAF and TSC mutations are particularly resistant to mTOR inhibitors^66–68^. However, combined inhibition of the AKT/mTOR and Wnt/β-catenin pathways has been shown to significantly improve the effectiveness of mTOR inhibitors^3,58^. Given that Ephexin1 is involved in both Ras/AKT and Wnt/β-catenin signaling, it may help overcome resistance to mTOR inhibitors. Studies in patients with renal cell carcinoma have shown that mutations in TSC1/2 and mTOR are associated with rapalog resistance, although these mutations are not present in most patients, supporting the potential of targeting Ephexin1 in overcoming resistance^68^.

In cancer treatment, drug side effects are also a major concern. Although mTOR inhibitors are generally well tolerated, they can cause serious side effects due to the essential role of mTOR in normal cellular homeostasis. For instance, in a phase 2 clinical trial of Voxtalisib (50 mg, twice daily), serious side effects were reported in 58.1% of patients^8^. Ephexin1, normally expressed at low levels in most tissues, is overexpressed in lung and colon cancer cells^29,30,33,34^. Therefore, reducing the dosage of mTOR inhibitors while effectively targeting Ephexin1 may offer a treatment strategy that reduces side effects while maintaining efficacy.

Translation of 5′-TOP motif-containing mRNAs is activated during cancer cell invasion, migration and metastasis, with RNA-binding proteins such as LARP1, CNBP, AUF1 and TIAR1 involved in this process^9,12–15^. However, the precise interactions among these proteins remain poorly understood. In our study, we found that Ephexin1 selectively regulates the translation of cancer-specific 5′-TOP mRNAs rather than ribosomal proteins, which are typical TOP mRNAs. While it is possible that Ephexin1 directly interacts with specific translation initiation factors to mediate this regulation, the evidence remains insufficient to confirm this mechanism. An alternative hypothesis is that Ephexin1 interacts with RNA-binding proteins to achieve selective translational control. Given the involvement of LARP1, CNBP, AUF1 and TIAR1 in 5′-TOP mRNA regulation and the largely unexplored nature of their regulatory networks, it is plausible that Ephexin1 coordinates with these proteins to modulate the translation of cancer-specific mRNAs. Furthermore, Ephexin1-mediated activation of mTOR specifically governs the activity of these RBPs in a context-dependent manner by regulating their post-translational modifications. Additionally, selective activation of these RBPs would result in Ephexin1-driven targeted translation of 5′-TOP mRNAs. Understanding how Ephexin1 interacts with these RNA-binding proteins could provide valuable insights into mRNA translation regulation in cancer cells and suggest new therapeutic targets to disrupt aberrant protein synthesis pathways involved in tumor growth.

In summary, our study highlights the critical role of Ephexin1 in cancer progression, particularly through its selective regulation of 5′-TOP mRNA translation. This identifies Ephexin1 as a key factor in mTOR-regulated gene expression and cancer pathogenesis (Fig. 6n). Given the enhanced efficacy of mTOR inhibitors when Ephexin1 is downregulated, Ephexin1 represents a promising target for synthetic lethality strategies in mTOR-targeted cancer therapies.

## Supplementary information

Supplementary Information

Supplementary Table
