# Supplementary material for: The role of Ephexin1 in translation and mTOR-targeted cancer therapy
Source: Exp Mol Med. 2025 Aug 25;57(8):1847–60. doi: 10.1038/s12276-025-01520-2 (PMC12411628; doi:10.1038/s12276-025-01520-2)
Supplement: Supplementary file 1 — Supplementary Information [file 12276_2025_1520_MOESM1_ESM.pdf]

List of Supplementary information for **'The role of Ephexin1 in translation and mTOR-targeted cancer therapy'**

Supplementary Figure Legends and Figures………………………………………p2-12

SUPPLEMENTARY FIGIRE LEGENDS AND FIGIRES

Supplementary Fig. 1

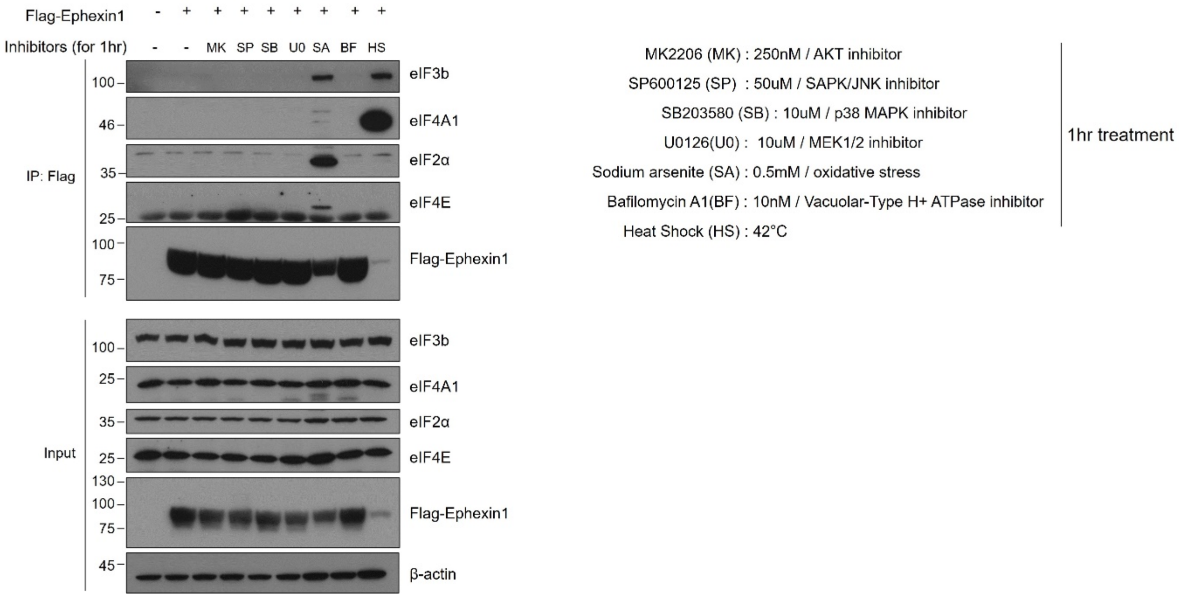

Supplementary Fig. 1 Arsenite and Heat-Shock treatment induce interaction between Ephexin1 and translation factors. HEK293T cells transfected with Flag-tagged Ephexin1 were treated with inhibitors or Heat_Shock (at 42°C) for 1 hr, followed by immunoprecipitation using anti-Flag antibody and western blot analysis using the indicated antibodies.

Supplementary Fig. 2

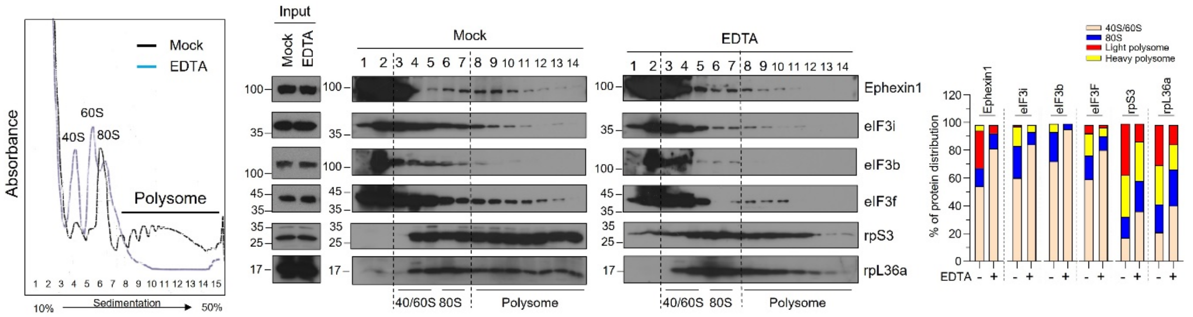

Supplementary Fig. 2 Disruption of the translation complex by EDTA causes a shift of Ephexin1 and translation initiation factors to the light fraction. Distribution of proteins after mock or EDTA treatment in polysome profiling analysis of H1299 cells.

Supplementary Fig. 3

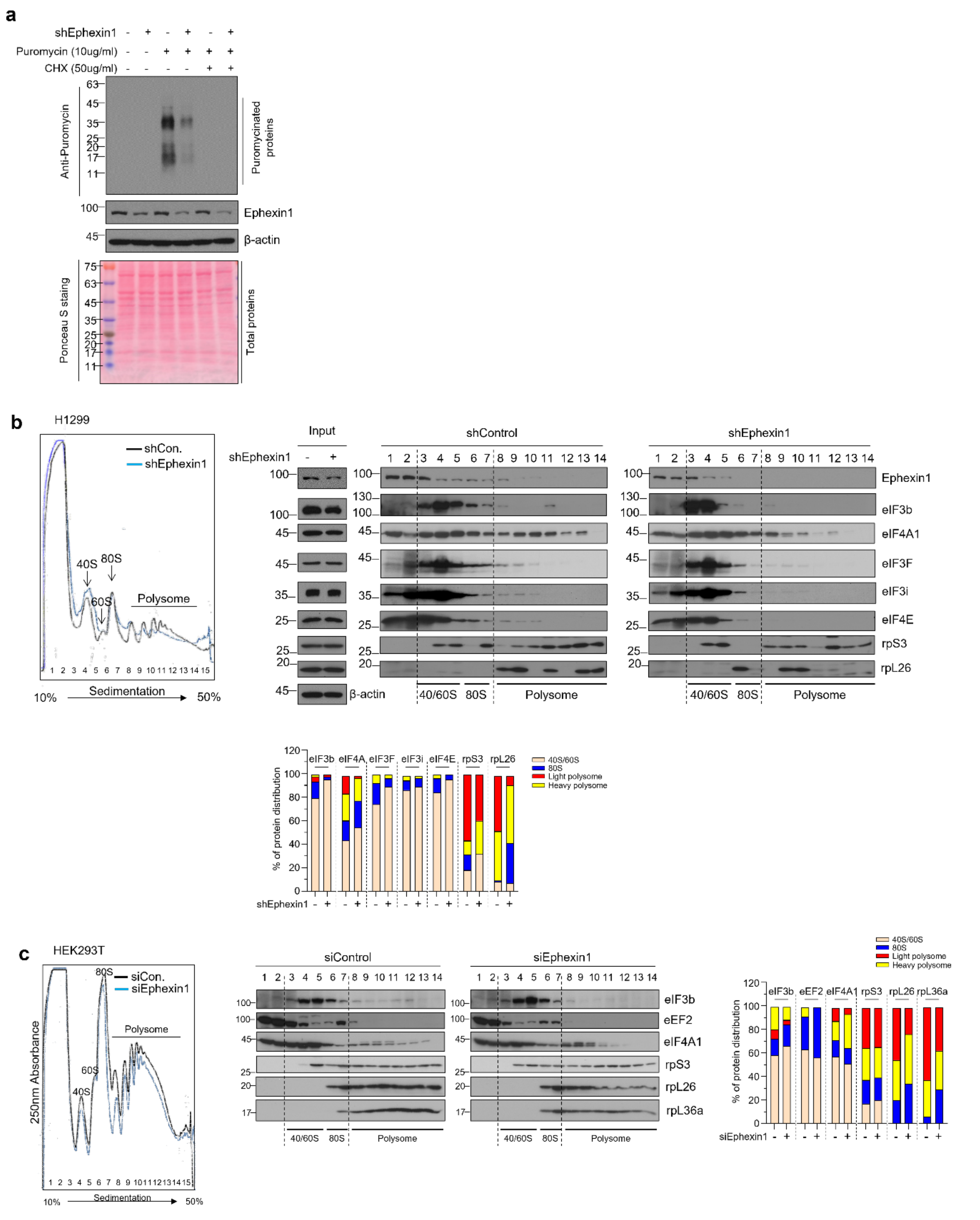

Supplementary Fig. 3 Deficiency of Ephexin1 causes inhibition of translation. a Ribo_puromycylation assay showing global translation profile of shControl or shEphexin1-H1299 cells. Puromycylated protein levels were assessed in cell lysates by immunoblotting with anti-Puromycin. Total protein levels are indicated by Ponceau S staining. **b** Protein

distribution in polysome profiling analysis of shControl or shEphexin1_H1299 cells. Western blot analysis was carried out with the indicated antibodies. **c** Protein distribution in polysome profiling analysis of siControl or siEphexin1_HEK293T cells. Western blot analysis was carried out with the indicated antibodies.

Supplementary Fig. 4

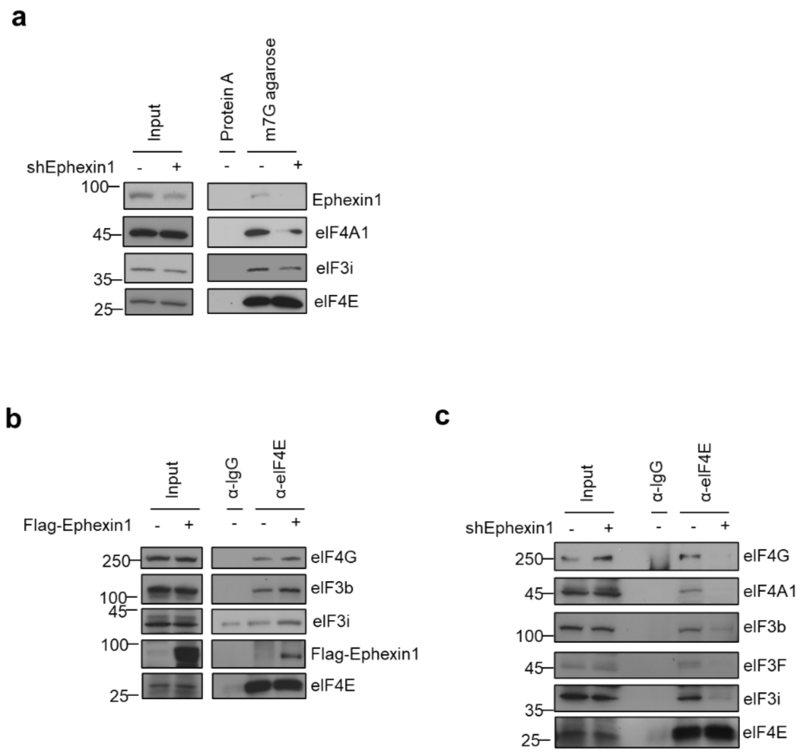

Supplementary Fig. 4 Ephexin1 enhances the formation of the translation initiation complex. a m7G pulldown analysis in control and Ephexin1-deficient HEK293T cell lines. Proteins associated with the m7G cap were identified through Western blot analysis. **b-c** Immunoprecipitation analysis using eIF4E antibody in Flag-Ephexin1 overexpressing or Ephexin1-deficient cells.

Supplementary Fig. 5

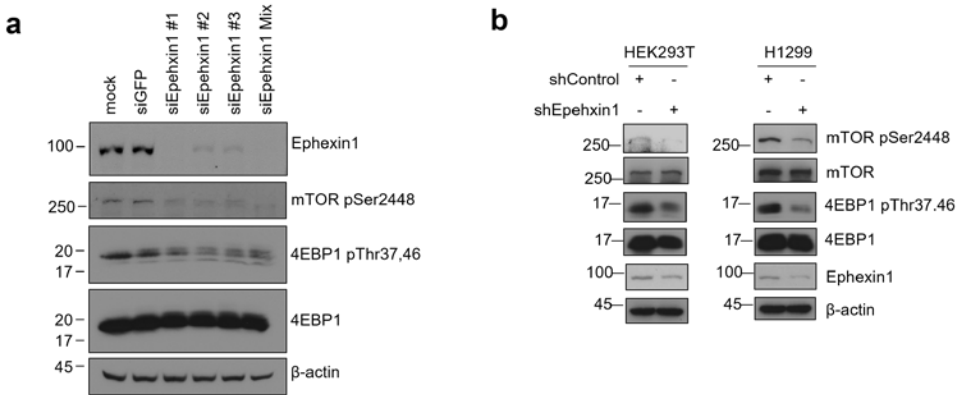

Supplementary Fig. 5 Deficiency of Ephexin1 inhibits translation. a H1299 cells were depleted of Epheixin1 using three Ephexin1 siRNAs, and the level of 4EBP1 phosphorylated protein was confirmed by western blot analysis. **b** shControl and shEphexin1 were transfected into HEK293T and H1299 cells for 96 h and analyzed by western blot analysis to detect protein levels.

Supplementary Fig. 6

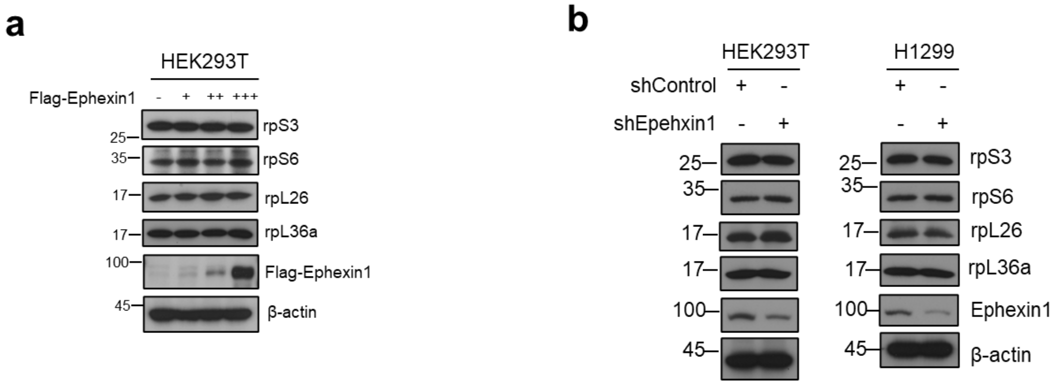

Supplementary Fig. 6 Ephexin1 does not regulate ribosomal proteins. a Quantitative changes in ribosomal proteins in response to Ephexin1 overexpression were assessed by Westrern blot. **b** shControl and shEphexin1 were transfected into HEK293T and H1299 cells for 96 h and analyzed by western blot analysis to detect protein levels.

Supplementary Fig. 7

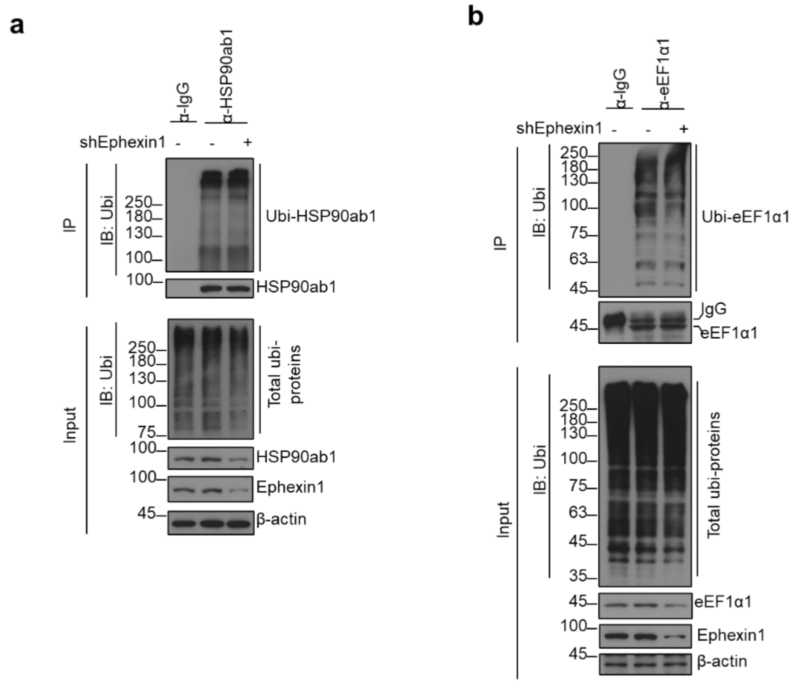

Supplementary Fig. 7 Ephexin1 deficiency does not affect ubiquitination of HSP90ab1 and eEF1α1. a-b Analysis of ubiquitination after immunoprecipitation using HSP90ab1 and eEF1a1 antibodies. Immunoblots were performed using the indicated antibodies.

Supplementary Fig. 8

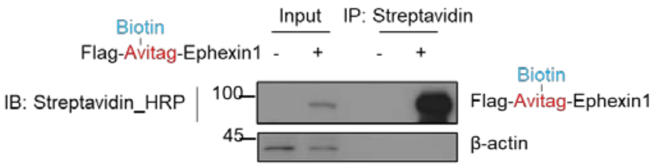

Supplementary Fig. 8 Ephexin1 deficiency does not affect ubiquitination of HSP90ab1 and eEF1α1. Immunoblot analysis to confirm RNP-IP after streptavidin pulldown in HEK293T/Flag-biotinylated-Ephexin1 cell line.

Supplementary Fig. 9

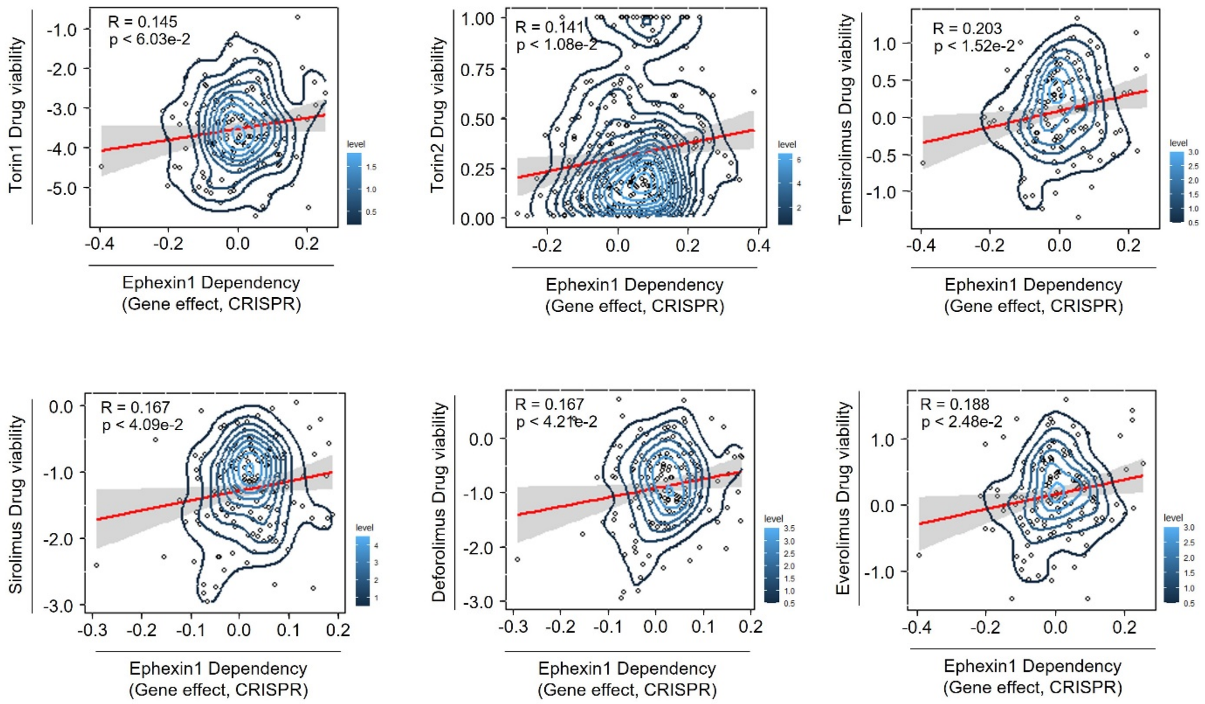

Supplementary Fig. 9 Positive correlation between mTOR inhibitor targeted drug sensitivity and Ephexin1. Correlation analysis between the gene effect of Ephexin1 (CRISPR, DEMETER2) and mTOR targeted agent sensitivity.

Supplementary Fig. 10

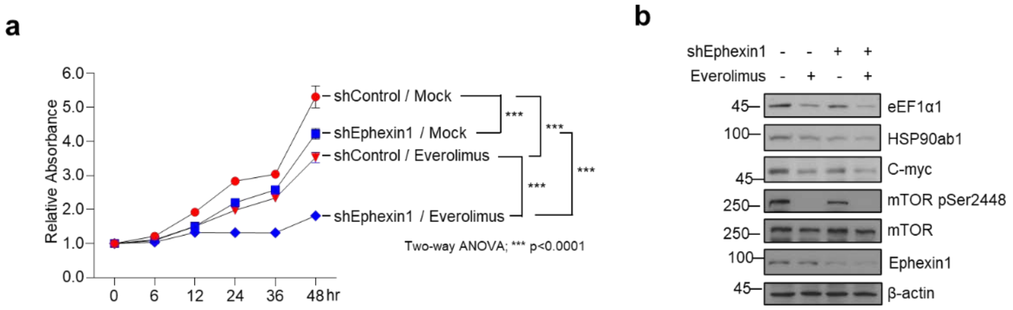

Supplementary Fig. 10 Ephexin1 deficiency enhances the therapeutic effect of Everolimus. **a** Cell survival was analyzed in Ephexin1-deficient H1299 cells and control cells treated with 20 nM Everolimus for 0–48 hours. **b** Control and Ephexin1-deficient H1299 cells treated with Everolimus (20nM for 12 hour) were analyzed.
